# Altered Gut Microbiota and Shift in *Bacteroidetes* between Young Obese and Normal-Weight Korean Children: A Cross-Sectional Observational Study

**DOI:** 10.1155/2020/6587136

**Published:** 2020-08-18

**Authors:** Saeam Shin, Ky Young Cho

**Affiliations:** ^1^Department of Laboratory Medicine, Yonsei University College of Medicine, Seoul 03722, Republic of Korea; ^2^Department of Pediatrics, Kangnam Sacred Heart Hospital, Hallym University College of Medicine, Seoul 07441, Republic of Korea

## Abstract

Emerging data suggest that the gut microbiome is related to the pathophysiology of obesity. This study is aimed at characterizing the gut microbiota composition between obese and normal-weight Korean children aged 5-13. We collected fecal samples from 22 obese and 24 normal-weight children and performed 16S rRNA gene sequencing using the Illumina MiSeq platform. The relative abundance of the phylum *Bacteroidetes* was lower in the obese group than in the normal-weight group and showed a significant negative correlation with BMI *z*-score. Linear discriminative analysis (LDA) coupled with effect size measurement (LEfSe) analysis also revealed that the *Bacteroidetes* population drove the divergence between the groups. There was no difference in alpha diversity, but beta diversity was significantly different between the normal-weight and obese groups. The gut microbial community was linked to BMI *z*-score; blood biomarkers associated with inflammation and metabolic syndrome; and dietary intakes of niacin, sodium, vitamin B6, and fat. The gut microbiota of the obese group showed more clustering of genera than that of the normal-weight group. Phylogenetic investigation of communities by reconstruction of unobserved states (PICRUSt) analysis revealed that the functions related to carbohydrate and lipid metabolism in the microbiota were more enriched in the normal-weight group than in the obese group. Our data may contribute to the understanding of the gut microbial structure of young Korean children in relation to obesity. These findings suggest that *Bacteroidetes* may be a potential therapeutic target in pediatric obesity.

## 1. Introduction

Childhood obesity is a major public health concern worldwide [1]. Obese children have a high risk of developing adult obesity and obesity-related comorbidities, including type 2 diabetes mellitus, cardiovascular disease, and psychological problems [2, 3]. Recently, accumulating evidence suggests that the human gut microbiota is associated with many chronic diseases, including obesity. Previous studies in adults have demonstrated that obese individuals have different microbial compositions than lean individuals [4, 5]. The gut microbiota is closely related to energy harvest and metabolism in humans [6, 7]. Therefore, microbiota-targeted strategies have attracted much attention in the context of obesity treatment [8, 9].

Studies on the obesity-related microbiota in children are still scarce. Gut microbial composition is known to vary with age, ethnicity, and diet [6, 10]. Although previous studies have suggested that the gut microbiota of infants is converted into adult-like composition in the first 1-3 years [11], some evidence indicates that the microbiota continues to change until adolescence [12]. Therefore, valuable information on the obesity-related microbiota could be obtained from different ethnic and age groups.

The objective of this study was to characterize the composition of the gut microbiota among obese and normal-weight Korean children. Amplicons of the 16S rRNA gene were sequenced using Illumina MiSeq to analyze the composition of the gut microbiota. The dietary and lifestyle patterns of the participants and the levels of blood biochemical markers related to inflammation and metabolic disease were measured and examined in relation to the gut microbiota composition. The correlation structure of the microbiota was shown using network analysis, and predictive functional differences between groups were identified by phylogenetic investigation of communities by reconstruction of unobserved states (PICRUSt) analysis.

## 2. Materials and Methods

### 2.1. Subjects, Questionnaire, and Anthropometric Measurements

Forty-six children who were 5-13 years of age were enrolled in the study at Hallym University Kangnam Sacred Heart Hospital from December 2017 to March 2018 (NCT03388411). This study protocol was approved by the Institutional Review Board of the Hallym University Kangnam Sacred Heart Hospital (2017-09-015). Written informed consent was obtained from all subjects of the study and their parents, in accordance with the Declaration of Helsinki. Based on the 2017 Korean growth chart [13], subjects with a body mass index (BMI) ≥ 95th percentile were classified as the obese group, and subjects with 5th percentile < BMI < 85th percentile were classified as the normal-weight group. For a month prior to the stool sampling, none of the subjects had taken antibiotics/probiotics/steroids or had diarrhea. None of the subjects had acute infections or chronic disease. Subjects completed questionnaires on lifestyle, bowel habits, and dietary intake and submitted them at the hospital visit. Anthropometric measurements, including height, weight, waist circumference, midarm circumference, hip circumference, thigh circumference, and blood pressure, were performed by professionally trained personnel [14, 15]. Body composition analysis including skeletal muscle mass and total body fat content was measured using the inBody 770 analyzer (Biospace Co. Ltd., Seoul, Korea).

### 2.2. Dietary Assessments

The children and their parents received specific training from a dietitian to describe in a proper way all the foods and the quantities consumed, including the name/brand of the consumed food, recipes of dishes, method of preparation or cooking, and portion sizes. After training with the dietitian, the participants filled out everything the subjects ate and drank for 3 days: 2 weekdays and 1 weekend day. Using data from the dietary records, nutrient intakes were calculated by a dietitian using the Computer-Aided Analysis Program 4.0 for professionals (CAN-pro 4.0, Korean Nutrition Society, Seoul, Korea).

### 2.3. Blood Sampling and Biochemical Analysis

After a 12-hour overnight fast, blood samples were taken from the subjects. The levels of glucose, aspartate aminotransferase (AST), alanine aminotransferase (ALT), insulin, total cholesterol, triglycerides, high-density lipoprotein- (HDL-) cholesterol, low-density lipoprotein- (LDL-) cholesterol, high-sensitivity C-reactive protein (hs-CRP), uric acid, iron, and unsaturated iron binding capacity (UIBC) were measured using a Hitachi 7600 autoanalyzer (Hitachi, Tokyo, Japan). Total iron binding capacity (TIBC) was calculated as the sum of the serum iron and UIBC levels. Transferrin saturation (Tf%) was calculated as (serum iron/(serum iron + UIBC)) × 100. Concentrations of ferritin, insulin, and 25-OH vitamin D were determined using an ADVIA Centaur XP (Siemens Healthcare Diagnostics, Deerfield, IL, USA). The complete blood count was analyzed by an ADVIA 2120i (Siemens Healthcare Diagnostics, Tarrytown, NY, USA). The neutrophil-to-lymphocyte ratio (NLR) was calculated as the ratio of the neutrophil count to the lymphocyte count. The hemoglobin A1c (HbA1c) level was determined using a D-100 system (Bio-Rad Laboratories, Hercules, CA, USA). Insulin resistance and beta-cell function were evaluated by the homeostasis model assessment methods (HOMA-IR and HOMA-%B, respectively). HOMA-IR was calculated as (insulin (*μ*IU/mL) × glucose (mg/dL))/405, and HOMA-%B was calculated as (20 × insulin (*μ*IU/mL))/(glucose (mg/dL)/18‐3.5).

### 2.4. Stool Sampling, Bacterial DNA Extraction, Illumina MiSeq Sequencing, and Bioinformatics

The stool samples were collected in sterile containers and immediately frozen at -80°C until DNA extraction. DNA was extracted using a QIAamp DNA Stool Mini Kit (Qiagen, Valencia, CA, USA) according to the manufacturer's instructions. Using 2 *μ*L of the extracted DNA, polymerase chain reaction (PCR) amplifications were performed with the primers targeting the V3 to V4 regions of the 16S rRNA gene [16]. The products were then amplified by the second PCR with index primers. Equal concentrations of amplicons were pooled together and purified using an AMPure bead kit (Agencourt Bioscience, Beverly, MA, USA). The product size and quality were assessed on an Agilent 2100 Bioanalyzer (Agilent Technologies, Palo Alto, CA, USA). Sequencing was carried out at ChunLab, Inc. (Seoul, Korea) using the Illumina MiSeq platform (Illumina, San Diego, CA, USA). The raw reads were checked for quality, and low-quality reads (average score < 25) were filtered by a Trimmomatic tool (version 0.32). Then, the paired-end sequences were merged using a PANDASeq Paired-end Assembler [17]. Chimeric sequences were removed using the UCHIME algorithm [18]. The taxonomic classification of each read was performed based on the EzBioCloud database (http://eztaxon-e.ezbiocloud.net) [19]. Sequences that corresponded to the reference sequence with greater than 97% similarity in EzBioCloud were considered to be identified at the species level. To compare the operational taxonomic units (OTUs) between samples, we determined shared OTUs through the EzBioCloud program (Chunlab Inc.). The functional potential of the microbiota was inferred using PICRUSt metagenomics prediction [20] and was categorized into levels 1-3 based on Kyoto Encyclopedia of Genes and Genomes (KEGG) pathways [21].

### 2.5. Statistical Analysis

All statistical analyses were carried out using R software (version 3.5.2, http://www.r-project.org/). Based on the Shapiro-Wilk normality test, data are presented as the means and standard deviations (for continuous variables with normal distribution) or medians and interquartile ranges (for continuous variables with skewed distribution). Categorical variables are expressed as frequencies and percentages. In analyzing the characteristics, nutrient intake, and blood biochemical profiles of the participants, the *t*-test or the Kruskal-Wallis Rank Sum test was used according to the results of the normality test. For comparison of the gut microbiota between groups, the Mann-Whitney *U* test was used for continuous variables, and chi-square was used for categorical variables. Correlations between continuous variables were calculated using the Pearson correlation test. All analyses were performed after normalizing for the copy number of the bacterial 16S rRNA gene. The ACE index, the number of observed OTUs, the Chao1 richness estimate, and the Jackknife estimate were used to compare gut microbiota richness between samples. The within sample (alpha) diversity was compared using the Simpson diversity index, the Shannon index, and phylogenetic diversity. Cluster analysis was performed with nonmetric multidimensional scaling (NMDS), after computing the Bray-Curtis dissimilarity between each pair of individuals. The Fast UniFrac analysis was used to calculate the (beta) diversity between groups and was visualized with a principal coordinate analysis (PCoA). Differences in beta diversity between the normal-weight and obese groups were tested with nonparametric analysis of variance based on 999 permutations (permutational multivariate analysis of variance (PERMANOVA)). The differently abundant bacterial taxa between the normal-weight and obese groups were identified using the linear discriminant analysis (LDA) coupled with effect size measurement (LEfSe) method [22]. To analyze the ability of specific taxa to predict obesity, we calculated the area under the receiver operating characteristic curve (AUC of ROC). Multivariate analysis for relationships between gut microbial community composition, BMI *z*-score, and blood biochemical markers was performed using canonical correspondence analysis (CCA). Using the R package qgraph [23], we performed a network analysis to identify the difference in the correlation network of the gut microbiota between the normal-weight and obese groups. To obtain the relative robustness, the sample coverage threshold for the identified genera was set at ≥0.5. The resulting *p* values were adjusted for multiple testing with the false discovery rate (FDR) method [24]. *p* values ≤ 0.05 were considered statistically significant.

## 3. Results

### 3.1. Participant Characteristics, Lifestyle Questionnaire, and Blood Biochemical Marker

A total of forty-six children were enrolled in this study (obese group: *n* = 22; normal-weight group: *n* = 24). The characteristics of the participants are summarized in Table 1. The sex distribution showed no difference between the normal-weight and obese groups (*p* = 0.853; Table 1). The individuals in the normal-weight group were slightly younger than those in the obese group (*p* = 0.048; Table 1). All anthropometric measurements, including height (*p* = 0.002), weight (*p* < 0.001), BMI (*p* < 0.001), BMI *z*-score (*p* < 0.001), waist circumference (*p* < 0.001), midarm circumference (*p* < 0.001), hip circumference (*p* < 0.001), and thigh circumference (*p* < 0.001), showed significantly higher values in the obese group than in the normal-weight group (Table 1). Systolic/diastolic blood pressure (*p* = 0.002/*p* < 0.001), skeletal muscle mass (*p* < 0.001), and total body fat content (*p* < 0.001) also showed significantly higher values in the obese group than in the normal-weight group (Table 1). The percentage of children born by cesarean section was significantly higher in the obese group (*p* = 0.012; Table 1). Questionnaires on lifestyle patterns differed between the normal-weight and obese groups. The percentage of children who did not exercise at all was 36.4% in the obese group and 0% in the normal-weight group (*p* = 0.004; Table 1). The percentage of children who exercised for 30 minutes or more per day was significantly higher in the normal-weight group than in the obese group (*p* = 0.025; Table 1). The percentage of children who watched television or used electronic devices (video games, smart phones, or computers) more than two hours a day was significantly higher in the obese group than in the normal-weight group (*p* = 0.017; Table 1). The percentage of children who used electronic devices near bedtime tended to be higher in the obese group than in the normal-weight group (*p* = 0.072; Table 1).

Analysis of energy and nutrient intakes from three-day dietary records showed significant differences in total energy (*p* < 0.001), protein (*p* < 0.001), fat (*p* = 0.001), carbohydrate (*p* = 0.028), cholesterol (*p* = 0.002), total fatty acid (*p* = 0.010), polyunsaturated fatty acid (*p* = 0.001), trace mineral (phosphorus, iron, sodium, potassium, and zinc (*p* < 0.05)), and vitamin (thiamine, niacin, vitamin B6, and vitamin E (*p* = 0.004)) intakes between the normal-weight and obese groups (Table 2).

Among the measured blood biochemical markers, glucose (*p* = 0.027), ALT (*p* = 0.018), triglycerides (*p* = 0.003), LDL-cholesterol (*p* = 0.021), hs-CRP (*p* < 0.001), uric acid (*p* = 0.032), ferritin (*p* < 0.001), insulin (*p* < 0.001), HOMA-IR (*p* < 0.001), HOMA-%B (*p* < 0.001), mean platelet volume (MPV) (*p* = 0.017), white blood cell (WBC) count (*p* = 0.039), neutrophil percentage (*p* = 0.003), and NLR (*p* = 0.024) were significantly higher in the obese group than in the normal-weight group (Table 3). On the other hand, HDL-cholesterol (*p* = 0.025), 25-OH vitamin D (*p* = 0.002), and lymphocyte percentage (*p* = 0.047) were significantly lower in the obese group than in the normal-weight group (Table 3).

### 3.2. Gut Microbial Composition and Diversity

After filtering low-quality, nontarget, and chimeric amplicons, 16S rRNA gene sequencing resulted in a total of 3.9 million high-quality reads from 46 fecal samples. The median sequencing read was 78,155 (quartiles: 69,929; 85,731). We obtained a median value of 412.5 OTUs per sample (316; 529.3) after excluding low-abundance OTUs (<1% of total).

**Figure 1:**
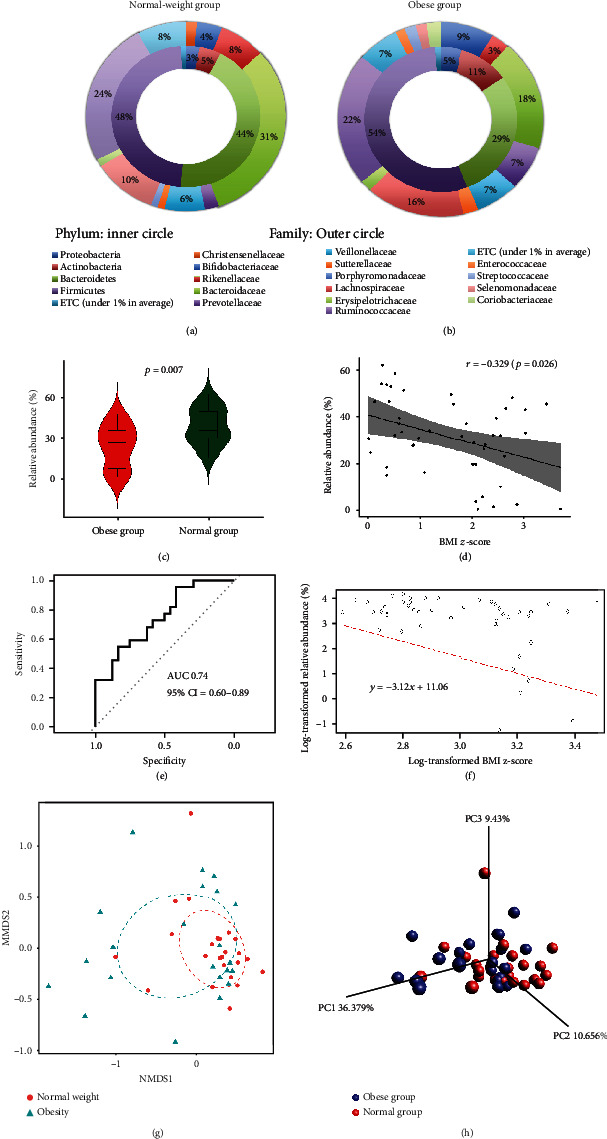
Gut microbiota composition in the normal-weight (a) and obese (b) groups. The inner circle shows the composition at the phylum level, and the outer circle shows the composition at the family level. Violin plot showing the median, spread, and distribution pattern of the relative abundance of *Bacteroidetes* between the obese group and the normal-weight group (c). Correlation scatter plot of the relative abundance (%) of *Bacteroidetes* and BMI *z*-scores of participants (d). Receiver operator characteristic (ROC) curve of the relative abundance of *Bacteroidetes* to predict obesity (e). The area under the curve (AUC) was 0.74 (95% CI: 0.60-0.89). Interaction between *Bacteroidetes* and BMI *z*-score (F). Cluster analysis was performed with NMDS analysis using Bray-Curtis distance (g). Principal coordinate analysis of the gut microbiota at the genus level according to the normal-weight (red) and obese (blue) groups (h). Principal components (PCs) 1, 2, and 3 explained 36.379%, 10.656%, and 9.43% of the variance, respectively (h). This result suggests that the gut microbial composition observed in the obese group was significantly different from that of the normal-weight group (*p* = 0.009, PERMANOVA on Fast UniFrac distances).

At the phylum level, the predominant bacterial taxa were *Firmicutes* and *Bacteroidetes*, followed by *Actinobacteria* and *Proteobacteria* in both groups (Figures 1(a) and 1(b)). The relative abundance of the phylum *Bacteroidetes* was significantly decreased in obese children (obese group: median 36.6 (0.3; 52.9); normal-weight group: 45.2 (10.5; 69.1)) (*p* = 0.007; Figure 1(c)). The relative abundance of *Bacteroidetes* was significantly negatively correlated with BMI*z*-score (Figure 1(d)). The ROC analysis of *Bacteroidetes* for predicting obesity showed good performance (AUC: 0.7443; 95% confidence interval (CI): 0.603-0.8856), unlike the analysis of *Firmicutes* (AUC: 0.5606; 95% CI: 0.3839-0.7373) (Figure 1(e)). Multivariate regression analysis showed that the log-transformed BMI *z*-score and the log-transformed relative abundance of major taxa belonging to the phylum *Bacteroidetes* were negatively associated after adjusting for age, sex, and delivery type (beta = −3.12, standard estimates = 11.06; Figure 1(f)). In the stepwise logistic regression model, the odds ratio for risk of obesity associated with *Bacteroidetes* at the phylum level was 0.87 after adjusting for age, sex, and delivery type (95% CI: 0.80-0.95; *p* = 0.003) (Table 4).

There was no significant difference in the relative abundance of *Firmicutes* (obese: 53.1 (45.1; 73.3); normal-weight: 45.7 (24.7; 76.6)), *Actinobacteria* (obese: 1.28 (0.56; 10.55); normal-weight: 0.89 (0.31; 2.99)), or *Proteobacteria* (obese: 8.5 (1.41; 15.0); normal-weight: 5.34 (1.72; 11.12)) (*p* = 0.053, *p* = 0.235, and *p* = 0.416, respectively; Supplementary Figures S1A, S1B, and S1C). The *Firmicutes-*to-*Bacteroidetes* (F : B) ratio was significantly elevated in obese children (obese: 1.5 (0.9; 18.4); normal-weight: 1.1 (0.4; 2.9)) (*p* = 0.012; Supplementary Figure S1D). Among family-level taxa, the relative abundance of *Lachnospiraceae* was significantly higher in the obese group than in the normal-weight group (obese: 13.49 (10.97; 18.24); normal-weight: 9.9 (7.52; 12.94)) (*p* = 0.022; Supplementary Figure S2A). Among taxa at the species level, the abundance of *Bacteroides ovatus* was significantly lower in the obese group than in the normal-weight group (obese: 0.07 (0.02; 1.04); normal-weight: 1.04 (0.67; 2.8)) (*p* = 0.022; Figure S2B). There was no difference in the abundance of the genus *Akkermansia* between the groups (obese: 0.01 (0; 0.05); normal-weight: 0.14 (0; 1.27)) (*p* = 0.356; Supplementary Figure S2C).

We analyzed the correlation between BMI *z*-score and the relative abundance of bacterial taxa (Table 5). The relative abundance of the phylum *Bacteroidetes* was negatively correlated with BMI *z*-score (Pearson's correlation coefficient *r* = −0.329, *p* = 0.026; Figure 1(d)). Furthermore, BMI *z*-score was negatively correlated with the abundance of *Bacteroidia* at the class level; *Bacteroidales* at the order level; and *Bacteroidaceae*, *Devosia_f*, *Leptotrichiaceae*, *Odoribacteraceae*, *Porphyromonadaceae*, *Rikenellaceae*, and *Staphylococcaceae* at the family level (Table 5). The relative abundance of the family *Lachnospiraceae* was positively correlated with BMI *z*-score (*p* = 0.05; Table 5). The relative abundance of the phylum *Firmicutes* was not significantly correlated with BMI *z*-score (*r* = 0.24, *p* = 0.1).

Gut microbiota richness measures showed no obvious difference between samples from normal-weight and obese children (ACE, *p* = 0.725; the number of observed OTUs, *p* = 0.692; the Chao1 richness estimate, *p* = 0.676; and the Jackknife estimate, *p* = 0.775). In addition, several alpha diversity estimates, including the Simpson diversity index (*p* = 0.262), the Shannon index (*p* = 0.272), and phylogenetic diversity (*p* = 0.982), were not significantly different between normal-weight and obese children. NMDS analysis revealed separation and clustering of the obese group from the normal-weight group along the NMDS1 axis, while naïve tended to cluster along NMDS2 (Figure 1(g)). We used the Fast UniFrac analysis to measure beta diversity. The PCoA plot of the microbiota from all individuals in the normal-weight and obese groups is shown in Figure 1(h). The beta diversity showed a statistically significant difference between normal-weight and obese children at the genus level (*p* = 0.009, PERMANOVA on Fast UniFrac distances; Figure 1(h)).

### 3.3. Taxonomic Differences in the Microbiota between Obese and Normal-Weight Children

**Figure 2:**
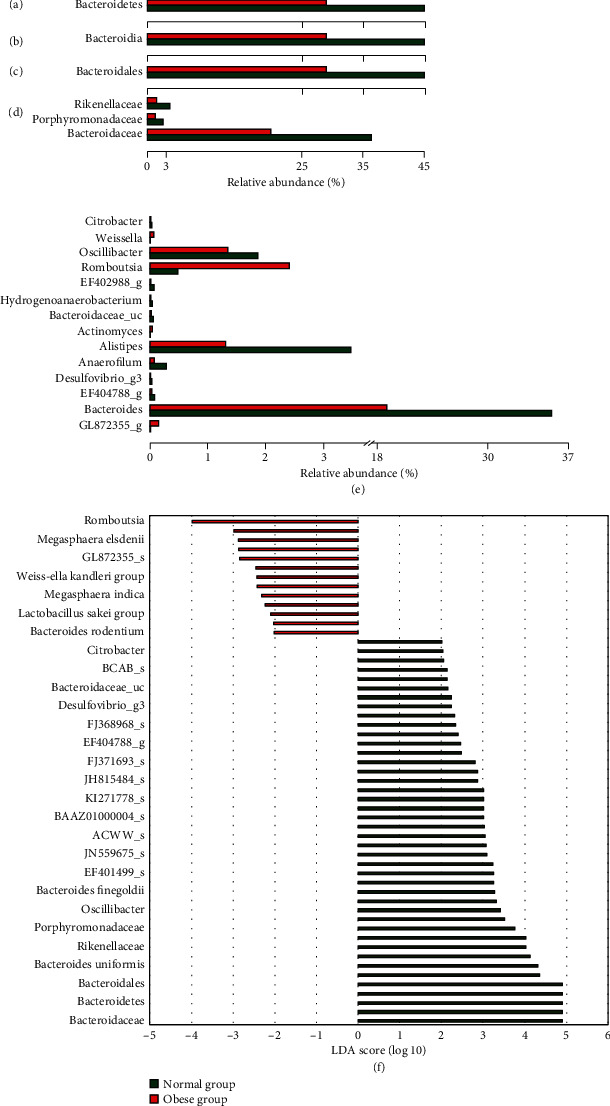
Linear discriminant analysis (LDA) coupled with effect size measurement (LEfSe) analysis identified the most differentially abundant taxa that distinguished the normal-weight group from the obese group. Comparison of relative abundance at the bacterial phylum (a), class (b), order (c), family (d), and genus € levels between the normal-weight and obese groups. LDA scores from LEfSe analysis, including the phylum, class, order, family, genus, and species levels (f). The enriched bacterial taxa of the normal-weight group are marked with green bars, and the taxa enriched in the obese group are indicated with red bars (f). Only taxa meeting an LDA significance threshold > 2 are shown (f).

To identify the specific microbial profile distinguishing obese and normal-weight children, a metagenomics biomarker discovery approach using the LEfSe method was applied to assess the effect size of each differently abundant taxon (Figure 2). The LDA effect size values are shown in Figure 2(f). Using the LEfSe method, we found that *Bacteroidetes* at the phylum level; *Bacteroidia* at the class level; *Bacteroidales* at the order level; *Bacteroidaceae*, *Porphyromonadaceae*, and *Rikenellaceae* at the family level; and *Bacteroides*, *EF404788_g*, *Desulfovibrio_g3*, *Anaerofilum*, *Alistipes*, *Bacteroidaceae_uc*, *Hydrogenoanaerobacterium*, *EF402988_g*, *Oscillibacter*, and *Citrobacter* at the genus level were significantly enriched in the normal-weight group (*p* < 0.05; Figure 2). This population is dominated by bacteria belonging to the *Bacteroidetes* phyla in the normal-weight group. In addition, *Actinomyces*, *Romboutsia*, *Weissella*, and *GL872355_g* at the genus level were significantly enriched in the obese group (*p* < 0.05; Figure 2).

### 3.4. Relationship between Gut Microbial Community Composition, BMI *z*-Score, Blood Biochemical Markers, and Dietary Intake

**Figure 3:**
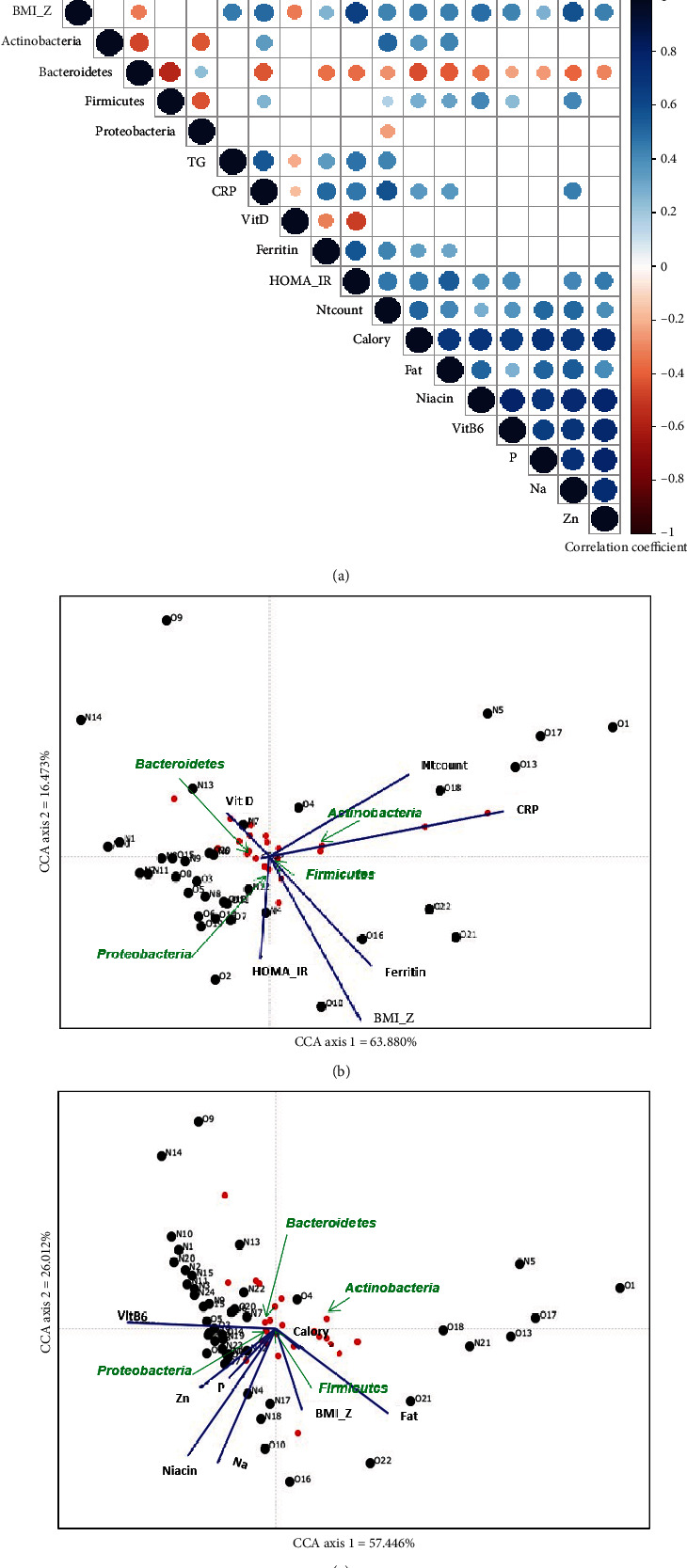
Correlation plot showing Pearson's correlation coefficient between blood biochemical markers related to insulin resistance and inflammation, major taxa, and dietary intake (a). Only statistically significant values are shown (*p* ≤ 0.05) (a). Positive correlations are displayed in blue, and negative correlations are displayed in red (a). Color intensity and the size of the circle are proportional to the correlation coefficients (a). Canonical correspondence analysis of gut microbial community composition at the phylum level with respect to BMI *z*-score, blood biochemical markers including 25-OH vitamin D, neutrophil count, CRP, ferritin, and HOMA-IR (b), or dietary intake including calories, fat, Na, niacin, Zn, P, and vitamin B6 (c). The blue lines indicate the direction and magnitude of variables associated with bacterial community composition (b, c). Red dots represent different bacterial phyla, and black dots represent each patient (O—obese group; N—normal-weight group) (b, c). Axes 1 and 2 can explain 80.4% of the data variance in the correlation between the microbiota and blood biomarkers associated with inflammation and metabolic syndrome (b). Axes 1 and 2 can explain 83.5% of the data variance in the correlation between microbiota and dietary intake (c). Abbreviations: VitD—25-OH vitamin D; Ntcount—neutrophil count; CRP—high-sensitivity C-reactive protein; BMI_Z—body mass index *z*-score; HOMA_IR—the homeostasis model assessment-estimated insulin resistance; Zn—zinc; TGs—triglycerides; VitB6—vitamin B6.

We generated a correlogram to visualize the degree of association between BMI *z*-score, major phyla, blood biochemical markers, and dietary intake of energy and nutrients (Figure 3(a)). Variables with highly significant differences between the two groups (*p* value ≤ 0.1) were selected from the univariate analysis. Variables with a correlation coefficient (*r*) of 0.8 or greater were regarded as the same variables, and then one representative variable was selected from the same variables. The BMI *z*-score showed a significant positive correlation with inflammatory markers, including HOMA-IR; neutrophil count; and serum levels of triglycerides, hs-CRP, and ferritin, and with increased dietary intake of calories, fat, niacin, vitamin B6, P, Na, and zinc (Figure 3(a)). On the other hand, serum vitamin D levels and the proportion of *Bacteroidetes* were negatively correlated with BMI *z*-score (Figure 3(a)). The *Actinobacteria* population showed negative correlations with the *Bacteroidetes* and *Proteobacteria* populations and positive correlations with blood markers including hs-CRP and neutrophil count and with dietary intake of calories and fat (Figure 3(a)). The correlogram showed that *Bacteroidetes* and *Firmicutes* exhibited different orientations in regard to correlation with most of the variables, including blood biomarkers and dietary intakes (Figure 3(a)). The *Bacteroidetes* population showed a negative correlation with the *Firmicutes* population and a positive correlation with the *Proteobacteria* population (Figure 3(a)). Moreover, the proportion of *Bacteroidetes* was negatively correlated with inflammatory markers, including hs-CRP, ferritin, HOMA-IR, and neutrophil count, and with dietary intake of calories, fat, niacin, vitamin B6, P, Na, and zinc (Figure 3(a)). On the other hand, the *Firmicutes* population showed a negative correlation with the proportion of *Proteobacteria* and positive correlations with the inflammatory markers hs-CRP and neutrophil count and with dietary intake of calories, fat, niacin, vitamin B6, and Na (Figure 3(a)).

We performed CCA to visualize the relationship between gut microbial community composition, BMI *z*-scores, and blood biochemical markers (Figure 3(b)) or dietary intake (Figure 3(c)). Variables were selected from the same standards with the correlogram. The distance between two points shows the significance of the correlation. The distance between *Bacteroidetes* and the microbial community in the normal-weight group is shorter than that between *Firmicutes* and the microbial community in the normal-weight group, suggesting that *Bacteroidetes* has a strong correlation with the normal-weight group (Figures 3(b) and 3(c)). The length of the blue line is proportional to the degree of importance. As shown in Figure 3(b), BMI *z*-score, HOMA-IR, hs-CRP, ferritin, and neutrophil count are more important in the microbial community of the obese group, whereas vitamin D is an important factor in the microbial community of the normal-weight group. Figure 3(c) shows that fat, Na, and niacin among dietary components, in addition to BMI *z*-score, are more important in the microbial community of the obese group, whereas vitamin B6 intake is an important factor in the microbial community of the normal-weight group. *Firmicutes* was negatively correlated with serum vitamin D levels and dietary intake of vitamin B6 (Figures 3(b) and 3(c)). *Bacteroidetes* was negatively correlated with BMI *z*-score, serum ferritin level, and fat intake (Figures 3(b) and 3(c)). *Actinobacteria* showed a negative correlation with HOMA-IR and dietary intake of Zn, P, niacin, and Na (Figures 3(b) and 3(c)). *Proteobacteria* was negatively correlated with neutrophil count and hs-CRP (Figures 3(b) and 3(c)).

### 3.5. Correlation Network

**Figure 4:**
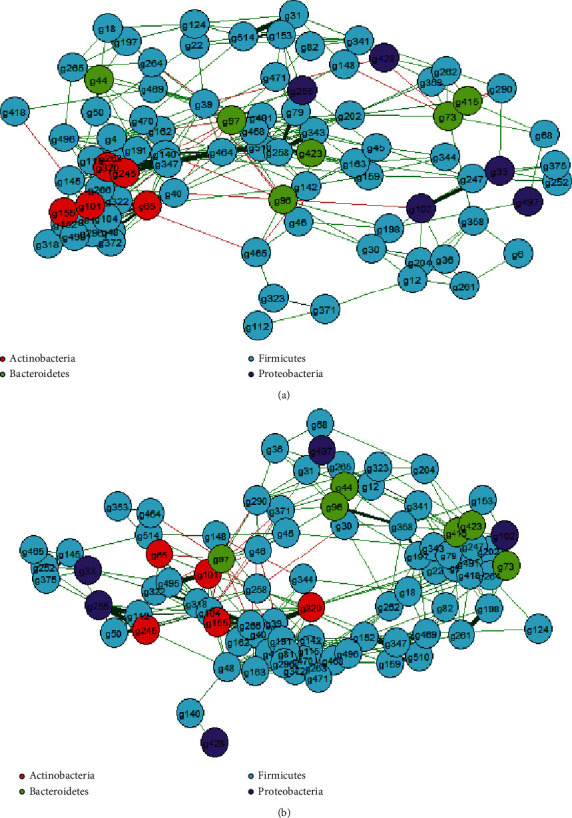
Correlation network analysis of the gut microbiome of the normal-weight (a) and obese (b) groups. Each node shows one genus, and nodes are colored by phylum affiliation. The color of the edges shows positive (green) and negative (red) pairwise correlations between genera. The color intensity of the edges indicates the relative strength of the correlation. Genera with sample coverage < 0.5 and correlations with a *p* value > 0.05 were removed.

We performed a correlation network analysis to investigate whether obesity was associated with alterations in the overall correlation structure of the gut microbiota (Figure 4 and Supplementary Table S1). Constructed networks revealed that samples from the normal-weight group had fewer edges, a lower mean degree, and a longer mean distance than those from the obese group, which indicates that there were fewer significant correlations and less clustering of genera (Supplementary Table S2). The betweenness centrality was higher in the obese group, which indicates that only a few genes were highly correlated in a network (Supplementary Table S2). *Bacteroidetes* showed higher positive intraphylum correlations in the normal-weight group, and *Firmicutes* showed higher positive intraphylum correlations in the obese group (Supplementary Table S2).

### 3.6. PICRUSt

**Figure 5:**
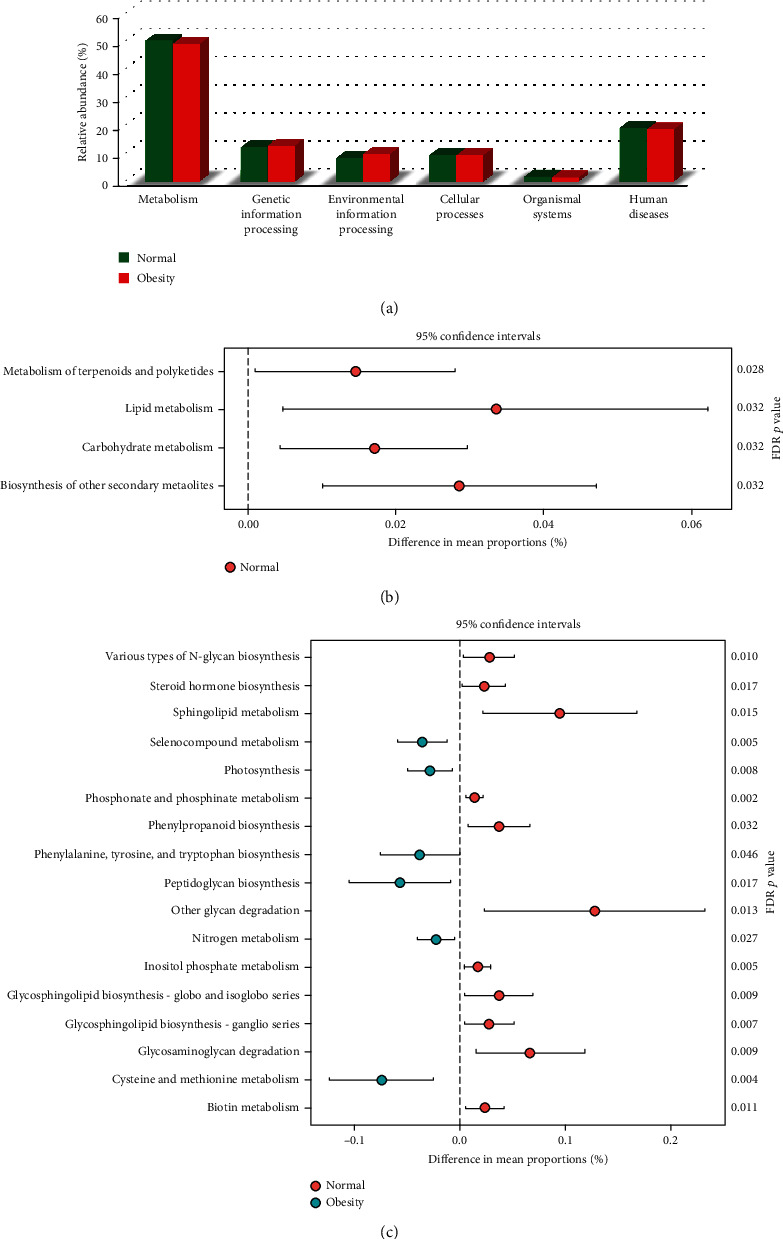
Distribution of tier 1 KEGG functional categories from the normal-weight (green) and obese (red) groups (a). Significant differences in the relative abundances of second (b) and third (c) tier KEGG functional categories between the normal-weight (orange) and obese (green) groups. Significance was determined using the Kruskal-Wallis test with FDR correction. Only functional categories with corrected *p* < 0.05 are shown.

To investigate the differences in microbial functions between the normal-weight and obese groups, we assessed the microbial community functional potential using PICRUSt analysis. The distribution of tier 1 KEGG functional categories was similar between the normal-weight and obese groups (Figure 5(a)). The largest number of genes (approximately 48%) corresponded to a function that encoded proteins involved in “metabolism” among tier 1 KEGG categories. Then, we examined which metabolic pathways in the tier 2 and tier 3 KEGG categories showed statistically significant differences between the normal-weight and obese groups (Supplementary Table S3). In the tier 2 KEGG categories, the microbiota of the normal-weight group was enriched in the functional abundance of “metabolism of terpenoids and polyketides” (*p* = 0.032), “lipid metabolism” (*p* = 0.032), “carbohydrate metabolism” (*p* = 0.028), and “biosynthesis of other secondary metabolites” (*p* = 0.032) (Figure 5(b)). In the tier 3 KEGG categories, several pathways were enriched in the gut microbiota of the normal-weight group: “biotin metabolism” (*p* = 0.011), “glycosaminoglycan degradation” (*p* = 0.009), “glycosphingolipid biosynthesis-ganglio series” (*p* = 0.007), “glycosphingolipid biosynthesis-globo and isoglobo series” (*p* = 0.009), “inositol phosphate metabolism” (*p* = 0.004), “other glycan degradation” (*p* = 0.013), “phenylpropanoid biosynthesis” (*p* = 0.032), “phosphonate and phosphinate metabolism” (*p* = 0.001), “sphingolipid metabolism” (*p* = 0.015), “steroid hormone biosynthesis” (*p* = 0.018), and “various types of n-glycan biosynthesis” (*p* = 0.009) (Figure 5(c)). In contrast, several functional pathways were enriched in the microbiota of the obese group: “cysteine and methionine metabolism” (*p* = 0.004); “peptidoglycan biosynthesis” (*p* = 0.017); “phenylalanine, tyrosine, and tryptophan biosynthesis” (*p* = 0.046); “photosynthesis” (*p* = 0.008); and “seleno-compound metabolism” (*p* = 0.004) (Figure 5(c)).

## 4. Discussion

The present study showed differences in gut microbial composition between young normal-weight and obese Korean children aged 5-13 years. Obese children showed a significant reduction in *Bacteroidetes*, an elevated F : B ratio, and significantly different beta diversity compared with the same parameters among normal-weight children, as described by previous studies [4, 25, 26]. The *Bacteroidetes* population was also detected by LEfSe with a high LDA score, suggesting that it is the key phylotype responsible for the differences between the normal-weight and obese groups. The relative abundance of *Firmicutes*, however, revealed no significant difference between the groups. The results of our study suggest the importance of *Bacteroidetes* in pediatric obesity. Recent evidence has indicated that *Bacteroidetes* is a potentially modifiable therapeutic target because it is more largely influenced by environmental factors rather than host genetics [4, 27, 28]. In the future, prospective intervention studies will be needed to explore the impact of the specific species or strains belonging to the *Bacteroidetes* phylum on pediatric obesity modulation. Family *Lachnospiraceae* was significantly correlated with BMI *z*-score. This result is consistent with a previous experimental result showing that the colonization of bacteria belonging to *Lachnospiraceae* induces the development of diabetes in germ-free *ob*/*ob* mice [29]. From these findings, it could be assumed that *Lachnospiraceae* is involved in the development of metabolic dysfunction in children. *Akkermansia* is a mucin-degrading bacterium, and its abundance has been reported to be negatively correlated with obesity in previous studies with adults [30]. In one study, *Akkermansia* was reduced in obese children aged 4-5 years living in Sweden, which was analyzed by quantitative PCR [31]. However, the current reports including our study, using 16S rRNA next generation sequencing analysis, revealed no significant difference in *Akkermansia* levels between normal-weight and obese children [32, 33]. This disparity can be explained with the differences in the methodology, ethnicity, and extent of its colonization which starts from early childhood and reaches a similar level to adults [34].

To investigate the relationships between gut microbial community composition, BMI, and selected variables from the biochemical markers and diet intake, CCA analysis was performed. The gut microbial community in the obesity group revealed a strong correlation with BMI *z*-score, which was in line with previous reports [35, 36]. Inflammatory markers, including hs-CRP, neutrophil count, and ferritin, were related to microbial composition in the obese group, suggesting that obesity is closely linked to inflammation [37]. Evidence for a relationship between inflammation and the microbiota continues to be revealed. Bacterial products, such as lipopolysaccharide and short-chain fatty acids (SCFAs), can induce inflammation through immune cell activation and fat accumulation in adipocytes [38, 39]. These findings suggest the role of the gut microbiota in the development of inflammation in the pathogenesis of pediatric obesity. Among dietary intakes, niacin, Na, and fat seemed to affect gut microbial composition in the obese group. Higher fat and Na intake is associated with obesity and metabolic syndrome [40, 41]. Moreover, a recent study indicated the possible association of chronic niacin overload on pediatric obesity [42]. In our study, dietary intake of vitamin B6 seemed to be important in the microbial community of normal-weight children. Prior work has suggested that the gut microbiota of lean adolescents seems to be more involved in vitamin B6 synthesis [25]. Further research is needed to corroborate the present results. The correlation network of the gut microbiota in this study showed that the normal-weight group had less clustering of genera than the obese group. This finding is consistent with prior studies by Riva et al. [32], showing that the gut microbiota in the obese group has a different correlation network structure than the gut microbiota in the normal-weight group.

The mechanisms by which the microbiota affects energy balance in the human body are not clear. Our results from the PICRUSt analysis showed that gut microbial function in obese children involves energy metabolism, such as photosynthesis and nitrogen metabolism, which can stimulate lipogenesis or gluconeogenesis [43]. Recent research has revealed that *Bacteroides ovatus* can release monosaccharides from cellulose and hemic cellulose for further metabolism by a wide variety of gut commensals via glycolytic pathways [44]. Our results showed that carbohydrate metabolism was more predicted in normal-weight children than in obese children, which can be speculated to be related to the positive association of *Bacteriodes ovatus* in normal-weight children. Our functional analysis also showed a significantly greater presentation of genes involved in amino acid metabolism, such as cysteine and methionine metabolism and tyrosine and phenylalanine biosynthesis, in obese children than in normal-weight children. The fermentation pathways of cysteine and methionine are included in sulfur metabolism, which leads to the production of hydrogen sulfide, which has been known to have detrimental effects on colonic epithelial energy metabolism [45, 46]. Tyrosine and phenylalanine biosynthesis are known to be associated with obesity, diabetes, and metabolic syndrome by reducing the activation of alkaline phosphatase [47, 48]. This result in our study may have important long-term implications for bowel health in the context of the consumption of excessive protein diets. In addition, higher abundances of microbial communities related to lipid metabolism were observed in normal-weight children than in obese children [49]. These changes in predicted metabolic pathways caused by intestinal microbiota can induce an imbalance between energy production and absorption. The specific mechanism associated with this functional analysis will need to be studied further.

In our study, hs-CRP, NLR, and MPV were higher in the obese group than in the normal-weight group. hs-CRP and NLR are well-known inflammatory markers that are associated with obesity because adipose tissue can be the major source of proinflammatory cytokines [50]. Increased MPV, a biomarker of platelet activity, is known to be associated with acute myocardial infarction, stroke, and thrombosis in individuals with morbid obesity [50]. The percentage of children born by cesarean section was significantly higher in the obese group than in the normal-weight group. This finding is the same as those of previous studies, which are explained by the disruption of mother-to-child transmission of gut microbiota associated with cesarean section [52, 53]. In regard to lifestyle, the use of electronic devices is becoming a major problem in the context of pediatric obesity rather than a lack of physical activity [54]. The present study also indicated that not only the lack of exercise but also the use of electronic devices for more than two hours a day was significantly higher in the obesity group. A recent study reported that electronic device usage close to bedtime can disrupt sleep patterns, which could lead to obesity [55]. Obesity is not only microbiota-driven; thus, a careful evaluation of all factors, including delivery mode, diet, and lifestyle, should be taken into account [56].

One of the strengths of this study is that it included only young Korean children, which makes the study population less influenced by ethnicity and environmental factors such as smoking, drugs, and alcohol use. Instead of simply comparing the microbiota between groups, we analyzed the relationship of multivariate factors in the microbiota, biochemical markers, and diet intake through a correlogram and CCA. Additionally, we showed the results of a network analysis and functional analysis of the gut microbiota in obese and normal-weight young children. The limitations of this study had to do with the small number of participants and the cross-sectional design, which prevents the determination of causality. Therefore, a prospective large-scale study is required to clarify the relationship between the microbiota and childhood obesity.

In conclusion, the microbial communities of obese children exhibited significant differences in beta diversity and a significantly elevated F : B ratio compared to those in normal-weight children. The phylum *Bacteroidetes* was significantly reduced in the obese group and was negatively correlated with BMI *z*-score. The LEfSe biomarker discovery analysis also suggested that the *Bacteroidetes* population was the key phylotype differentiating the two groups. These findings suggest the importance of *Bacteroidetes* in pediatric obesity. The gut microbial community in the obese group was linked to BMI *z*-score; blood biomarkers associated with inflammation and metabolic syndrome; and intakes of niacin, Na, and fat. In the microbial network analysis, the gut microbiota in the obese group showed more clustering of genera than the gut microbiota in the normal-weight group. PICRUSt analysis revealed that the functions related to carbohydrate and lipid metabolism were more enriched in the microbiota of the normal-weight group than in that of the obese group. Our data may contribute to the understanding of the gut microbial structure of young Korean children in relation to obesity. Further studies are required to target *Bacteroidetes* as a new therapeutic intervention for pediatric obesity.

## Figures and Tables

**Table 1:** Characteristics of the study participants.^∗^.

	Normal-weight (*n* = 24)	Obese (*n* = 22)	*p* ^†^
Sex (male, *n* %)	18 (75.0%)	15 (68.2%)	0.853
Age (years)	8.1 ± 1.5	9 ± 1.5	**0.048**
Anthropometric measurements			
Weight (kg)	27.3 (24.9; 30.1)	46.4 (42.0; 52.9)	**<0.001**
Weight (*z*-score)	0.7 ± 0.5	2.2 ± 0.5	**<0.001**
Height (cm)	129.5 ± 9.4	139.1 ± 10.8	**0.002**
Height (*z*-score)	0.6 ± 0.5	1 ± 0.7	**0.035**
BMI^†^ (kg/m^2^)	16.6 (15.5; 17.6)	24.4 (22.9; 25.7)	**<0.001**
BMI (*z*-score)	0.6 (0.4; 0.9)	2.5 (2.1; 2.8)	**<0.001**
Systolic blood pressure	98.5 (90.0; 111.5)	110.0 (100.0; 120.0)	**0.002**
Diastolic blood pressure	60.0 (60.0; 63.5)	70.0 (60.0; 70.0)	**<0.001**
Waist circumference (cm)	58.5 (55.5; 61.8)	77.8 (74.0; 81.5)	**<0.001**
Waist-to-height ratio	0.5 ± 0	0.6 ± 0	**<0.001**
Midarm circumference (cm)	20.3 ± 2.7	26.7 ± 2.8	**<0.001**
Hip circumference (cm)	69.9 ± 6.7	86.3 ± 8.0	**<0.001**
Thigh circumference (cm)	39.0 (35.6; 41.1)	46.0 (44.5; 51.5)	**<0.001**
Total body fat content (%)	22.1 (19.6; 26.4)	39.2 (33.8; 40.2)	**<0.001**
Skeletal muscle mass (kg)	10.2 (9.3; 11.3)	16.4 (13.9; 19.1)	**<0.001**
Delivery type			**0.012**
Vaginal : cesarean	18 (75.0%) : 6 (25.0%)	7 (33.3%) : 14 (66.7%)	
Lifestyle pattern			
Study time after school			0.094
≤1 hour	7 (29.2%)	1 (5.0%)	
>1 hour	17 (70.8%)	19 (95.0%)	
Exercise during the day			0.004
Yes	24 (100.0%)	14 (63.6%)	
No	0 (0.0%)	8 (36.4%)	
Exercise time during the day			0.025
≤30 min	2 (8.3%)	9 (40.9%)	
>30 min	22 (91.7%)	13 (59.1%)	
Exercise time during the day			0.219
≤1 hour	11 (45.8%)	15 (68.2%)	
>1 hour	13 (54.2%)	7 (31.8%)	
Time spent watching TV or using electronic devices			0.017
≤2 hours	20 (83.3%)	10 (45.5%)	
>2 hours	4 (16.7%)	12 (54.5%)	
Use electronic device for more than an hour before sleeping			0.072
Yes	4 (16.7%)	10 (45.5%)	
No	20 (83.3%)	12 (54.5%)	

^∗^Data are expressed as the mean ± standard deviation, median (interquartile range), or a number (%). ^†^Significant *p* values are shown in bold. Abbreviations: AST—aspartate aminotransferase.

**Table 2:** Energy and nutrient intake of the participants.^∗^.

	Normal-weight (*n* = 24)	Obese (*n* = 22)	*p* ^†^
Energy (kCal)	1543.4 ± 375.9	2027.9 ± 405.5	**<0.001**
Protein (g)	56.3 ± 18.4	82.0 ± 23.2	**<0.001**
Fat (g)	40.9 ± 14.0	62.8 ± 25.1	**0.001**
Carbohydrates (g)	240.6 ± 62.4	284.9 ± 68.4	**0.028**
Ca (mg)	486.5 ± 229.6	505.7 ± 277.9	0.801
P (mg)	889.3 ± 316.5	1173.7 ± 405.0	**0.011**
Iron (mg)	10.2 ± 4.4	15.0 ± 5.0	**0.002**
Na (mg)	2726.6 ± 1099.2	4105.3 ± 1073.9	**<0.001**
K (mg)	2076.5 ± 757.0	2566.7 ± 781.4	**0.039**
Zinc (mg)	8.1 ± 2.5	11.0 ± 2.8	**0.001**
Folic acid (*μ*g)	430.1 ± 229.6	448.9 ± 186.6	0.767
*β*-Carotene (*μ*g)	2430.8 (1691.5; 3646.6)	3350.5 (1979.1; 5071.1)	0.260
Retinol (*μ*g)	100.1 (69.1; 199.6)	157.5 (97.3; 305.2)	0.104
Thiamine (mg)	1.0 ± 0.3	1.4 ± 0.5	**0.002**
Riboflavin (mg)	1.2 ± 0.5	1.4 ± 0.6	0.296
Niacin (mg)	11.3 ± 5.0	18.2 ± 6.7	**<0.001**
Vitamin B6 (mg)	1.0 (0.9; 1.4)	1.6 (1.3; 2.0)	**0.001**
Vitamin C (mg)	95.4 (40.3; 226.9)	87.6 (52.2; 123.5)	0.532
Vitamin D (*μ*g)	2.6 (1.5; 6.0)	3.1 (2.0; 4.9)	0.891
Vitamin E (mg)	11.8 ± 5.0	18.1 ± 8.0	**0.004**
Fiber (g)	15.7 (11.8; 19.2)	16.9 (16.0; 18.8)	0.191
Cholesterol (mg)	250.1 ± 149.3	431.3 ± 224.9	**0.002**
Total fatty acid (mg)	23.8 ± 8.4	37.4 ± 21.0	**0.010**
Saturated fatty acid (mg)	9.7 (6.6; 12.1)	12.1 (6.0; 19.9)	0.094
Polyunsaturated fatty acid (mg)	6.0 ± 2.9	11.1 ± 5.5	**0.001**

^∗^Data are expressed as the mean ± standard deviation, median (interquartile range), or a number (%). ^†^Significant *p* values are shown in bold.

**Table 3:** Blood biochemical profiles of the participants.^∗^.

	Normal weight (*n* = 24)	Obese (*n* = 22)	*p* ^†^
Glucose (mg/dL)	97.2 ± 4.8	102 ± 6.7	**0.027**
AST (IU/L)	29.0 (25.0; 33.0)	25.0 (22.0; 26.0)	**0.027**
ALT (IU/L)	13.5 (13.0; 19.0)	19.5 (16.0; 29.0)	**0.018**
Total cholesterol (mg/dL)	168 ± 24.1	180 ± 25.1	0.165
Triglycerides (mg/dL)	47.5 (43.0; 67.0)	81.0 (61.0; 132.0)	**0.003**
HDL-cholesterol (mg/dL)	63.5 ± 11.3	54 ± 12.1	**0.025**
LDL-cholesterol (mg/dL)	94 ± 19.4	111.9 ± 22.9	**0.021**
hs-CRP (mg/L)	0.3 (0.2; 0.4)	2.1 (0.7; 3.1)	**<0.001**
Uric acid (mg/L)	4.2 ± 0.8	5 ± 1.3	**0.032**
Iron (*μ*g/dL)	104.4 ± 45.3	92 ± 33.7	0.353
TIBC (*μ*g/dL)	330.4 ± 27.3	347.2 ± 32.2	0.115
Transferrin saturation (%)	31.4 ± 12.5	26.6 ± 9.9	0.212
25-OH vitamin D (ng/mL)	15 ± 4.5	10.8 ± 3.2	**0.002**
Ferritin (ng/mL)	33.6 ± 14.3	62.8 ± 26.8	**<0.001**
Insulin (*μ*U/mL)	4.9 (3.8; 6.8)	13.8 (9.6; 18.6)	**<0.001**
HOMA-IR	1.2 (0.9; 1.8)	3.5 (2.4; 4.4)	**<0.001**
HOMA-%B	51.5 (43.0; 66.4)	122.4 (98.8; 172.9)	**<0.001**
Hemoglobin (g/dL)	13.4 ± 0.9	13.3 ± 0.8	0.955
HbA1c (%)	5.2 (5.1; 5.5)	5.3 (5.2; 5.5)	0.188
Platelet count (×10^3^/*μ*L)	312.8 ± 53	325.1 ± 67.7	0.567
MPV (fL)	7.2 (6.7; 7.3)	7.4 (7.1; 8.0)	**0.017**
PDW (%)	43.0 (41.2; 51.2)	48.0 (44.2; 50.4)	0.115
WBC count (×10^3^/*μ*L)	6 ± 1.1	7 ± 1.5	**0.039**
Neutrophil count	2.6 ± 0.7	3.6 ± 1.0	**0.003**
Neutrophil (%)	43.1 ± 9.2	50.6 ± 8.1	**0.015**
Lymphocyte count (×10^3^/*μ*L)	2.7 ± 0.7	2.8 ± 0.9	0.911
Lymphocyte (%)	45.2 ± 8.6	39.2 ± 8.6	**0.047**
Monocyte count (×10^3^/*μ*L)	0.3 ± 0.1	0.3 ± 0.1	0.519
Eosinophil count (×10^3^/*μ*L)	0.2 ± 0.1	0.2 ± 0.1	0.853
Basophil count (×10^3^/*μ*L)	0.0 (0.0; 0.0)	0.0 (0.0; 0.0)	0.474
Neutrophil-to-lymphocyte ratio	0.8 (0.7; 1.2)	1.2 (1.0; 1.7)	**0.024**

^∗^Data are expressed as the mean ± standard deviation, median (interquartile range), or a number (%). ^†^Significant *p* values are shown in bold. Abbreviations: AST—aspartate aminotransferase; ALT—alanine aminotransferase; HDL—high-density lipoprotein; LDL—low-density lipoprotein; hs-CRP—high-sensitivity C-reactive protein; TIBC—total iron binding capacity; MPV—mean platelet volume; PDW—platelet distribution width; WBC—white blood cell.

**Table 4:** Association between taxa and risk of obesity analyzed by logistic regression.

Level	Taxa	Adjusted odds ratio^∗^	95% CI	*p*
Phylum	*Bacteroidetes*	0.87	0.80-0.95	0.003
Class	*Bacteroidia*	0.87	0.80-0.95	0.003
Order	*Bacteroidales*	0.87	0.80-0.95	0.003
Family	*Bacteroidaceae*	0.90	0.84-0.97	0.004
Genus	*Bacteroides*	0.90	0.84-0.97	0.004

^∗^Adjusted for age, sex, and delivery type. Abbreviation: CI—confidence interval.

**Table 5:** Bacterial taxa correlated with BMI *z*-score.

Level	Taxa	*r* ^∗^	*p* ^∗∗^
Phylum	*Bacteroidetes*	-0.329	0.026
Class	*Bacteroidia*	-0.329	0.026
Order	*Bacteroidales*	-0.329	0.026
Family	*Bacteroidaceae*	-0.354	0.016
	*Devosia_f*	-0.405	0.005
	*Lachnospiraceae*	0.291	0.05
	*Leptotrichiaceae*	-0.314	0.034
	*Odoribacteraceae*	-0.317	0.032
	*Porphyromonadaceae*	-0.302	0.041
	*Rikenellaceae*	-0.377	0.01
	*Staphylococcaceae*	-0.311	0.035
	*AB559589_g*	0.304	0.04
	*Acetatifactor*	0.353	0.016
	*Acetitomaculum*	0.351	0.017
	*Acidaminococcus*	0.298	0.044
	*Alistipes*	-0.377	0.01
	*Anaerobium*	0.455	0.001
	*Anaerofilum*	-0.468	0.001
	*Anaerotruncus*	-0.38	0.009
	*Bacillus*	-0.294	0.047
	*Bacteroides*	-0.354	0.016
	*Brevundimonas*	-0.343	0.019
	*Catenibacterium*	0.315	0.033
	*Christensenellaceae_uc*	-0.324	0.028
	*Coprobacter*	-0.292	0.049
	*Desulfovibrio_g3*	-0.395	0.007
	*Devosia*	-0.405	0.005
	*Dielma*	-0.355	0.015
	*EF404788_g*	-0.364	0.013
	*Eubacterium_g21*	-0.313	0.034
	*FJ881296_g*	0.363	0.013
	*FN436026_g*	-0.308	0.037
	*GL872355_g*	0.294	0.048
	*Holdemania*	-0.325	0.027
	*Hydrogenoanaerobacterium*	-0.353	0.016
	*JPZU_g*	-0.295	0.047
	*KE159600_g*	-0.347	0.018
	*Rikenellaceae_uc*	-0.394	0.007
	*Senegalimassilia*	0.355	0.016
	*Staphylococcus*	-0.311	0.035
Species	*AB506430_s*	-0.315	0.033
	*ACWW_s*	-0.488	0.001
	*AF371599_s*	0.384	0.009
	*AM500802_g_uc*	-0.313	0.034
	*AY986255_s*	-0.305	0.039
	*Allisonella histaminiformans*	0.314	0.034
	*Anaerobium_uc*	0.389	0.008
	*Anaerotruncus colihominis*	-0.372	0.011
	*Atopostipes suicloacalis*	-0.33	0.025
	*BCAB_s*	-0.304	0.04
	*Bacteroides finegoldii*	-0.377	0.01
	*Bacteroides oleiciplenus*	-0.322	0.029
	*Bacteroides ovatus*	-0.322	0.029
	*Bacteroides uniformis*	-0.345	0.019
	*Butyricicoccus_uc*	0.323	0.029
	*Caproiciproducens_uc*	-0.34	0.021
	*Catenibacterium mitsuokai*	0.315	0.033
	*Clostridium_g12_uc*	0.348	0.018
	*Clostridium_g6_uc*	0.317	0.032
	*Corynebacterium pseudodiphtheriticum group*	0.328	0.026
	*DQ805799_s*	-0.341	0.02
	*DQ807741_s*	0.352	0.017
	*DQ905770_s*	-0.306	0.038
	*Desulfovibrio acrylicus group*	-0.395	0.007
	*Dielma fastidiosa*	-0.355	0.015
	*EF025278_g_uc*	0.37	0.011
	*EF400498_s*	-0.346	0.018
	*EF401207_s*	0.338	0.022
	*EF402071_s*	-0.394	0.007
	*EF404788_s*	-0.344	0.019
	*EF404944_s*	-0.308	0.038
	*EF405506_s*	-0.322	0.029
	*EF406456_s*	0.363	0.013
	*EF640143_s*	0.348	0.018
	*FJ368968_s*	-0.363	0.013
	*FJ371693_s*	-0.331	0.024
	*FJ505998_s*	-0.361	0.014
	*FJ681675_s*	-0.366	0.012
	*FJ825526_s*	0.314	0.034
	*FJ880315_s*	0.312	0.035
	*FN436026_s*	-0.308	0.037
	*Fusobacterium hwasookii*	0.304	0.04
	*Fusobacterium nucleatum group*	0.305	0.039
	*Gordonibacter pamelaeae*	-0.311	0.035
	*HM123979_g_uc*	0.338	0.022
	*HM124219_s*	0.331	0.025
	*HQ716480_s*	-0.321	0.03
	*HQ789817_s*	-0.371	0.011
	*HQ810970_s*	-0.34	0.021
	*JPZU_g_uc*	-0.302	0.042
	*JRNC_s*	0.305	0.039
	*KE159600_s*	-0.347	0.018
	*Klebsiella oxytoca group*	0.353	0.016
	*LARM_s*	-0.303	0.041
	*Lachnoanaerobaculum orale group*	-0.355	0.015
	*Megasphaera_uc*	0.337	0.022
	*PAC000196_s*	-0.389	0.008
	*PAC000740_s*	0.323	0.029
	*PAC000748_s*	-0.368	0.012
	*Parabacteroides_uc*	-0.319	0.031
	*Pseudogracilibacillus_uc*	0.322	0.029
	*Romboutsia sedimentorum*	0.387	0.008
	*Roseburia_uc*	0.386	0.008
	*Senegalimassilia anaerobia*	0.355	0.016

^∗^Pearson's correlation coefficient. ^∗∗^Taxa with *p* values > 0.05 were omitted.

## Data Availability

All raw 16S rRNA gene sequencing data were deposited in the NCBI Sequence Read Archive (SRA) under accession number SUB7560699 (BioProject PRJNA637782). Data related to the current study are available from the corresponding author on reasonable request.

## Supplementary Materials

Supplementary Materials Supplementary Figure S1: box plots for comparisons of *Firmicutes* (a), *Actinobacteria* (b), *Proteobacteria* (c), and *Firmicutes*-to-*Bacteroidetes* (F : B) ratio (d) between the normal and obese groups. There were no significant differences in the relative abundances of *Firmicutes*, *Actinobacteria*, or *Proteobacteria*. The F : B ratio revealed a significant difference between the normal and obese groups (*p* = 0.012). Supplementary Figure S2: differences in the relative abundance of the family *Lachnospiraceae* (a), the species *Bacteroides* ovatus (b), and the genus *Akkermansia* (c) between the normal and obese groups. Supplementary Table S1: name and classification of the genera shown in Figure 4. Supplementary Table S2: properties of correlation networks generated from the normal and obese groups. Supplementary Table S3: PICRUSTt predicted functions of KEGG categories presented in the obese group compared to those in the normal group.
