# Supplementary material for: Altered Gut Microbiota and Shift in Bacteroidetes between Young Obese and Normal-Weight Korean Children: A Cross-Sectional Observational Study
Source: Biomed Res Int. 2020 Aug 18;2020:6587136. doi: 10.1155/2020/6587136 (PMC7450312; doi:10.1155/2020/6587136)
Supplement: Supplementary Materials — Supplementary Figure S1: box plots for comparisons of Firmicutes (a), Actinobacteria (b), Proteobacteria (c), and Firmicutes-to-Bacteroidetes (F : B) ratio (d) between the normal and obese groups. There were no significant differences in the relative abundances of Firmicutes, Actinobacteria, or Proteobacteria. The F : B ratio revealed a significant difference between the normal and obese groups (p = 0.012). Supplementary Figure S2: differences in the relative abundance of the family Lachnospiraceae (a), the species Bacteroides ovatus (b), and the genus Akkermansia (c) between the normal and obese groups. Supplementary Table S1: name and classification of the genera shown in Figure 4. Supplementary Table S2: properties of correlation networks generated from the normal and obese groups. Supplementary Table S3: PICRUSTt predicted functions of KEGG categories presented in the obese group compared to those in the normal group. [file 6587136.f1.docx]

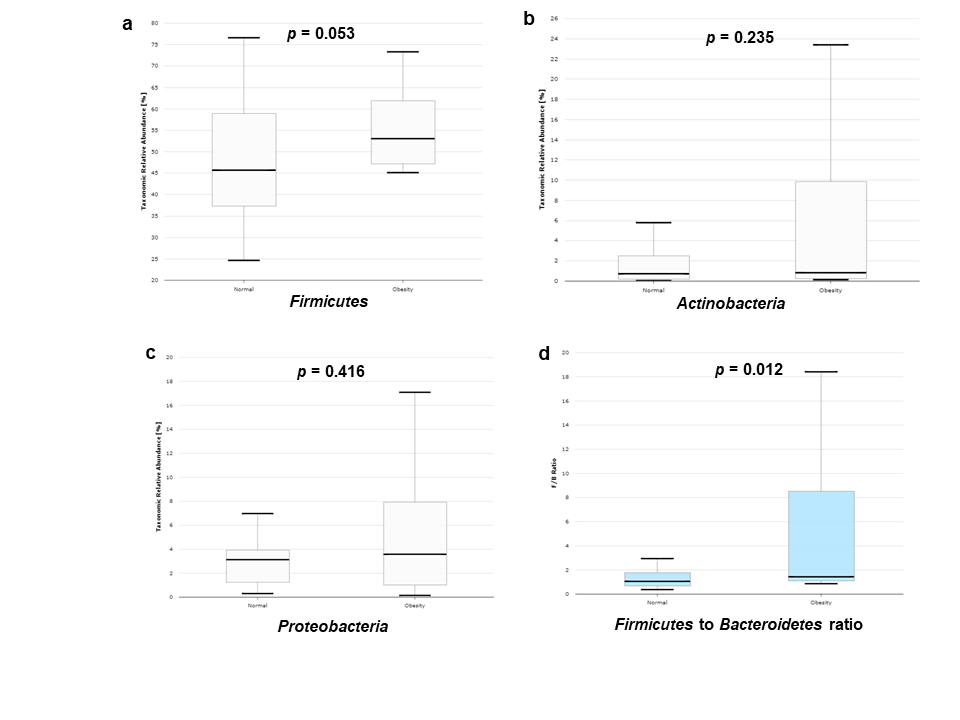


**Supplementary Figure S1.** Box plots for comparisons of *Firmicutes* (a), *Actinobacteria* (b), *Proteobacteria* (c), and *Firmicutes*-to-*Bacteroidetes* (F:B) ratio (d) between the normal and obese groups. There were no significant differences in the relative abundances of *Firmicutes*, *Actinobacteria*, or *Proteobacteria*. The F:B ratio revealed a significant difference between the normal and obese groups (*p* = 0.012).

**Supplementary Figure S2.** Differences in the relative abundance of the family *Lachnospiraceae* (a), the species *Bacteroides ovatus* (b), and the genus *Akkermansia* (C) between the normal and obese groups.


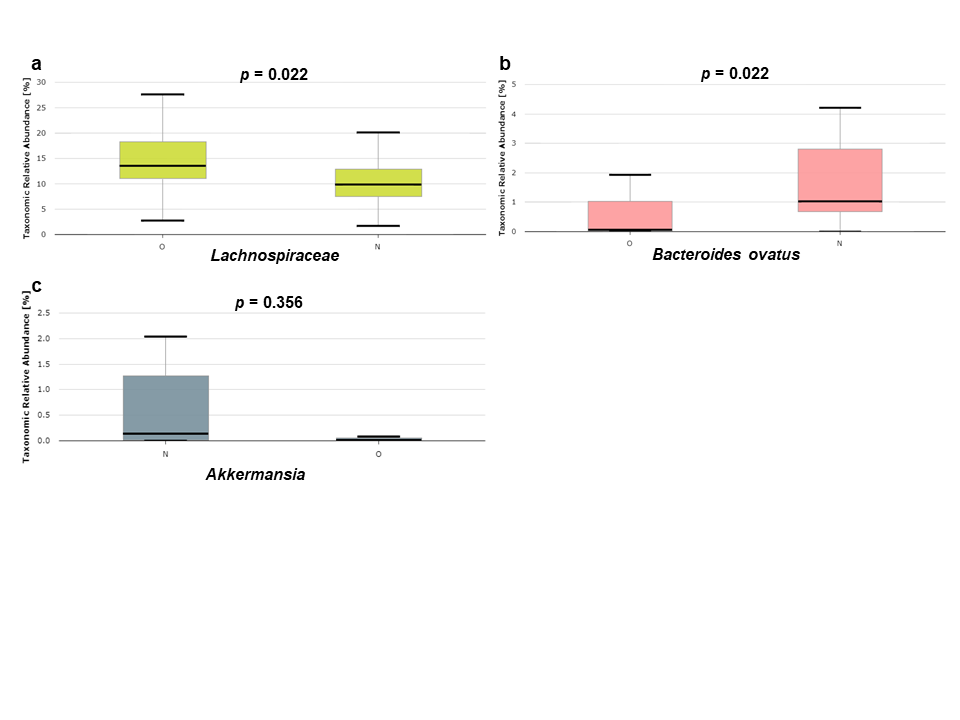


| **Supplementary Table S1.** Name and classification of the genera shown in Figure 4 | | | | | |
| --- | --- | --- | --- | --- | --- |
| Coding | Genus | Phylum | Class | Order | Family |
| g101 | *Bifidobacterium* | *Actinobacteria* | *Bifidobacteriales* | *Bifidobacteriaceae* | *Bifidobacterium* |
| g102 | *Bilophila* | *Proteobacteria* | *Desulfovibrionaceae* | *Bilophila* |  |
| g104 | *Blautia* | *Firmicutes* | *Clostridia* | *Clostridiales* | *Lachnospiraceae* |
| g112 | *Butyricicoccus* | *Firmicutes* | *Clostridia* | *Clostridiales* | *Ruminococcaceae* |
| g115 | *CCMM_g* | *Firmicutes* | *Erysipelotrichi* | *Erysipelotrichales* | *Erysipelotrichaceae* |
| g12 | *AB506319_g* | *Firmicutes* | *Clostridia* | *Clostridiales* | *Lachnospiraceae* |
| g124 | *Caproiciproducens* | *Firmicutes* | *Clostridia* | *Clostridiales* | *Ruminococcaceae* |
| g140 | *Clostridium* | *Firmicutes* | *Clostridia* | *Clostridiales* | *Clostridiaceae* |
| g142 | *Clostridium_g12* | *Firmicutes* | *Clostridia* | *Clostridiales* | *Lachnospiraceae* |
| g145 | *Clostridium_g21* | *Firmicutes* | *Clostridia* | *Clostridiales* | *Lachnospiraceae* |
| g148 | *Clostridium_g24* | *Firmicutes* | *Clostridia* | *Clostridiales* | *Lachnospiraceae* |
| g152 | *Clostridium_g6* | *Firmicutes* | *Erysipelotrichi* | *Erysipelotrichales* | *Erysipelotrichaceae* |
| g153 | *Clostridium_g7* | *Firmicutes* | *Clostridia* | *Clostridiales* | *Lachnospiraceae* |
| g155 | *Collinsella* | *Actinobacteria* | *Coriobacteriales* | *Coriobacteriaceae* | *Collinsella* |
| g159 | *Coprobacillus* | *Firmicutes* | *Erysipelotrichi* | *Erysipelotrichales* | *Erysipelotrichaceae* |
| g162 | *Coprococcus_g1* | *Firmicutes* | *Clostridia* | *Clostridiales* | *Lachnospiraceae* |
| g163 | *Coprococcus_g2* | *Firmicutes* | *Clostridia* | *Clostridiales* | *Lachnospiraceae* |
| g18 | *Dialister* | *Firmicutes* | *Negativicutes* | *Veillonellales* | *Veillonellaceae* |
| g191 | *Dorea* | *Firmicutes* | *Clostridia* | *Clostridiales* | *Lachnospiraceae* |
| g197 | *EF401862_g* | *Firmicutes* | *Clostridia* | *Clostridiales* | *Christensenellaceae* |
| g198 | *EF402988_g* | *Firmicutes* | *Clostridia* | *Clostridiales* | *Lachnospiraceae* |
| g202 | *EF404788_g* | *Firmicutes* | *Clostridia* | *Clostridiales* | *Ruminococcaceae* |
| g204 | *EF406589_g* | *Firmicutes* | *Clostridia* | *Clostridiales* | *Lachnospiraceae* |
| g22 | *EU381776_g* | *Firmicutes* | *Clostridia* | *Clostridiales* | *Mogibacterium_f* |
| g245 | *Eggerthella* | *Actinobacteria* | *Coriobacteriales* | *Coriobacteriaceae* | *Eggerthella* |
| g247 | *Eisenbergiella* | *Firmicutes* | *Clostridia* | *Clostridiales* | *Lachnospiraceae* |
| g252 | *Enterococcus* | *Firmicutes* | *Bacilli* | *Lactobacillales* | *Enterococcaceae* |
| g255 | *Escherichia* | *Proteobacteria* | *Enterobacteriaceae* | *Escherichia* |  |
| g258 | *Eubacterium_g1* | *Firmicutes* | *Erysipelotrichi* | *Erysipelotrichales* | *Erysipelotrichaceae* |
| g261 | *Eubacterium_g17* | *Firmicutes* | *Clostridia* | *Clostridiales* | *Lachnospiraceae* |
| g262 | *Eubacterium_g20* | *Firmicutes* | *Clostridia* | *Clostridiales* | *Lachnospiraceae* |
| g263 | *Eubacterium_g21* | *Firmicutes* | *Clostridia* | *Clostridiales* | *Lachnospiraceae* |
| g264 | *Eubacterium_g23* | *Firmicutes* | *Clostridia* | *Clostridiales* | *Ruminococcaceae* |
| g265 | *Eubacterium_g4* | *Firmicutes* | *Clostridia* | *Clostridiales* | *Lachnospiraceae* |
| g266 | *Eubacterium_g5* | *Firmicutes* | *Clostridia* | *Clostridiales* | *Lachnospiraceae* |
| g290 | *Faecalibacterium* | *Firmicutes* | *Clostridia* | *Clostridiales* | *Ruminococcaceae* |
| g296 | *Fusicatenibacter* | *Firmicutes* | *Clostridia* | *Clostridiales* | *Lachnospiraceae* |
| g30 | *AJ576336_g* | *Firmicutes* | *Clostridia* | *Clostridiales* | *Lachnospiraceae* |
| g31 | *AJ576354_g* | *Firmicutes* | *Clostridia* | *Clostridiales* | *Lachnospiraceae* |
| g318 | *Gemella* | *Firmicutes* | *Bacilli* | *Bacillales* | *Gemella_f* |
| g320 | *Gordonibacter* | *Actinobacteria* | *Coriobacteriales* | *Coriobacteriaceae* | *Gordonibacter* |
| g322 | *Granulicatella* | *Firmicutes* | *Bacilli* | *Lactobacillales* | *Aerococcaceae* |
| g323 | *HM123979_g* | *Firmicutes* | *Clostridia* | *Clostridiales* | *Lachnospiraceae* |
| g33 | *Haemophilus* | *Proteobacteria* | *Pasteurellaceae* | *Haemophilus* |  |
| g341 | *Holdemania* | *Firmicutes* | *Erysipelotrichi* | *Erysipelotrichales* | *Erysipelotrichaceae* |
| g343 | *Hungatella* | *Firmicutes* | *Clostridia* | *Clostridiales* | *Lachnospiraceae* |
| g344 | *Hydrogenoanaerobacterium* | *Firmicutes* | *Clostridia* | *Clostridiales* | *Ruminococcaceae* |
| g347 | *Intestinibacter* | *Firmicutes* | *Clostridia* | *Clostridiales* | *Peptostreptococcaceae* |
| g353 | *JPZU_g* | *Firmicutes* | *Clostridia* | *Clostridiales* | *Lachnospiraceae* |
| g358 | *KE159538_g* | *Firmicutes* | *Clostridia* | *Clostridiales* | *Lachnospiraceae* |
| g36 | *LLKB_g* | *Firmicutes* | *Clostridia* | *Clostridiales* | *Lachnospiraceae* |
| g371 | *Lachnospira* | *Firmicutes* | *Clostridia* | *Clostridiales* | *Lachnospiraceae* |
| g372 | *Lachnospiraceae_uc* | *Firmicutes* | *Clostridia* | *Clostridiales* | *Lachnospiraceae* |
| g375 | *Lactobacillus* | *Firmicutes* | *Bacilli* | *Lactobacillales* | *Lactobacillaceae* |
| g39 | *AM500802_g* | *Firmicutes* | *Clostridia* | *Clostridiales* | *Mogibacterium_f* |
| g4 | *AB185816_g* | *Firmicutes* | *Clostridia* | *Clostridiales* | *Ruminococcaceae* |
| g40 | *Murimonas* | *Firmicutes* | *Clostridia* | *Clostridiales* | *Lachnospiraceae* |
| g415 | *Odoribacter* | *Bacteroidetes* | *Bacteroidia* | *Bacteroidales* | *Odoribacteraceae* |
| g418 | *Oscillibacter* | *Firmicutes* | *Clostridia* | *Clostridiales* | *Ruminococcaceae* |
| g423 | *Parabacteroides* | *Bacteroidetes* | *Bacteroidia* | *Bacteroidales* | *Porphyromonadaceae* |
| g428 | *Parasutterella* | *Proteobacteria* | *Sutterellaceae* | *Parasutterella* |  |
| g44 | *Prevotella* | *Bacteroidetes* | *Bacteroidia* | *Bacteroidales* | *Prevotellaceae* |
| g45 | *Pseudoflavonifractor* | *Firmicutes* | *Clostridia* | *Clostridiales* | *Ruminococcaceae* |
| g46 | *AY305316_g* | *Firmicutes* | *Clostridia* | *Clostridiales* | *Lachnospiraceae* |
| g464 | *Romboutsia* | *Firmicutes* | *Clostridia* | *Clostridiales* | *Peptostreptococcaceae* |
| g465 | *Roseburia* | *Firmicutes* | *Clostridia* | *Clostridiales* | *Lachnospiraceae* |
| g468 | *Ruminococcaceae_uc* | *Firmicutes* | *Clostridia* | *Clostridiales* | *Ruminococcaceae* |
| g469 | *Ruminococcus* | *Firmicutes* | *Clostridia* | *Clostridiales* | *Ruminococcaceae* |
| g470 | *Ruminococcus_g2* | *Firmicutes* | *Clostridia* | *Clostridiales* | *Ruminococcaceae* |
| g471 | *Ruminococcus_g4* | *Firmicutes* | *Clostridia* | *Clostridiales* | *Lachnospiraceae* |
| g481 | *Sellimonas* | *Firmicutes* | *Clostridia* | *Clostridiales* | *Lachnospiraceae* |
| g491 | *Sporobacter* | *Firmicutes* | *Clostridia* | *Clostridiales* | *Ruminococcaceae* |
| g495 | *Streptococcus* | *Firmicutes* | *Bacilli* | *Lactobacillales* | *Streptococcaceae* |
| g496 | *Subdoligranulum* | *Firmicutes* | *Clostridia* | *Clostridiales* | *Ruminococcaceae* |
| g497 | *Sutterella* | *Proteobacteria* | *Sutterellaceae* | *Sutterella* |  |
| g50 | *Terrisporobacter* | *Firmicutes* | *Clostridia* | *Clostridiales* | *Peptostreptococcaceae* |
| g510 | *Turicibacter* | *Firmicutes* | *Erysipelotrichi* | *Erysipelotrichales* | *Erysipelotrichaceae* |
| g514 | *Veillonella* | *Firmicutes* | *Negativicutes* | *Veillonellales* | *Veillonellaceae* |
| g6 | *AB239481_g* | *Firmicutes* | *Clostridia* | *Clostridiales* | *Christensenellaceae* |
| g65 | *Actinomyces* | *Actinobacteria* | *Actinomycetales* | *Actinomycetaceae* | *Actinomyces* |
| g68 | *Agathobacter* | *Firmicutes* | *Clostridia* | *Clostridiales* | *Lachnospiraceae* |
| g73 | *Alistipes* | *Bacteroidetes* | *Bacteroidia* | *Bacteroidales* | *Rikenellaceae* |
| g79 | *Anaerofilum* | *Firmicutes* | *Clostridia* | *Clostridiales* | *Ruminococcaceae* |
| g81 | *Anaerostipes* | *Firmicutes* | *Clostridia* | *Clostridiales* | *Lachnospiraceae* |
| g82 | *Anaerotruncus* | *Firmicutes* | *Clostridia* | *Clostridiales* | *Ruminococcaceae* |
| g96 | *Bacteroidaceae_uc* | *Bacteroidetes* | *Bacteroidia* | *Bacteroidales* | *Bacteroidaceae* |
| g97 | *Bacteroides* | *Bacteroidetes* | *Bacteroidia* | *Bacteroidales* | *Bacteroidaceae* |

| **Supplementary Table S2.** Properties of correlation networks generated from the normal and obese groups | | |
| --- | --- | --- |
| Parameter | Normal | Obese |
| Nodes | 87 | 87 |
| Edges | 390 | 417 |
| Mean degree | 8.965517 | 9.586207 |
| Average path lengths | 2.938519 | 2.847902 |
| Assortativity | 0.5698669 | 0.4210643 |
| Transitivity | 0.5797567 | 0.5786072 |
| Betweenness centrality | 0.09690873 | 0.1619136 |
| Closeness centrality | 0.2460825 | 0.2414087 |
| Mean distance | 2.938519 | 2.847902 |
| Percent positive correlation in network | 0.392408 | 0.396685 |
| *Bacteroidetes* (intraphylum correlation, positive/negative) | 0.61, 190/311 | 0.58, 184/317 |
| *Firmicutes* (intraphylum correlation, positive/negative) | 0.67, 994/1491 | 0.70, 1025/1460 |

| **Supplementary Table S3**. PICRUSTt predicted functions of KEGG categories presented in the obese group compared to those in the normal group | | |  |
| --- | --- | --- | --- |
| KO functional categories |  | Difference between means (95% CI) obese vs normal | FDR *p* value |
| Level 2 | Level 3 |  |  |
| Global and overview maps | Biosynthesis of antibiotics | -0.142 (-0.298, 0.015) | 0.034 |
| Carbohydrate metabolism | Inositol phosphate metabolism | 0.017 (0.004, 0.03) | 0.005 |
| Energy metabolism | Photosynthesis | -0.028 (-0.049, -0.007) | 0.008 |
|  | Nitrogen metabolism | -0.022 (-0.04, -0.004) | 0.027 |
| Lipid metabolism | Steroid hormone biosynthesis | 0.023 (0.003, 0.044) | 0.017 |
|  | Glycerophospholipid metabolism | -0.018 (-0.036, 0.001) | 0.046 |
|  | Sphingolipid metabolism | 0.095 (0.022, 0.169) | 0.015 |
| Nucleotide metabolism | Purine metabolism | -0.086 (-0.176, 0.005) | 0.034 |
|  | Pyrimidine metabolism | -0.08 (-0.157, -0.004) | 0.054 |
| Amino acid metabolism | Cysteine and methionine metabolism | -0.074 (-0.123, -0.024) | 0.004 |
|  | Valine, leucine and isoleucine degradation | 0.025 (0, 0.05) | 0.027 |
|  | Lysine biosynthesis | -0.026 (-0.053, 0.001) | 0.051 |
|  | Phenylalanine, tyrosine and tryptophan biosynthesis | -0.038 (-0.075, -0.001) | 0.046 |
| Metabolism of other amino acids | Phosphonate and phosphinate metabolism | 0.014 (0.006, 0.022) | 0.002 |
|  | Selenocompound metabolism | -0.035 (-0.059, -0.012) | 0.005 |
| Glycan biosynthesis and metabolism | Other glycan degradation | 0.129 (0.024, 0.233) | 0.013 |
|  | Various types of N-glycan biosynthesis | 0.028 (0.003, 0.052) | 0.01 |
|  | Glycosaminoglycan degradation | 0.067 (0.015, 0.119) | 0.009 |
|  | Peptidoglycan biosynthesis | -0.057 (-0.105, -0.009) | 0.017 |
|  | Glycosphingolipid biosynthesis - globo and isoglobo series | 0.037 (0.005, 0.069) | 0.009 |
|  | Glycosphingolipid biosynthesis - ganglio series | 0.028 (0.005, 0.052) | 0.007 |
| Metabolism of cofactors and vitamins | Pantothenate and CoA biosynthesis | -0.022 (-0.043, 0) | 0.054 |
|  | Biotin metabolism | 0.024 (0.005, 0.042) | 0.011 |
| Metabolism of terpenoids and polyketides | Polyketide sugar unit biosynthesis | 0.013 (-0.005, 0.03) | 0.049 |
|  | Biosynthesis of enediyne antibiotics | 0.002 (0, 0.003) | 0.032 |
| Biosynthesis of other secondary metabolites | Phenylpropanoid biosynthesis | 0.038 (0.009, 0.067) | 0.032 |
|  | Flavone and flavonol biosynthesis | 0.005 (0.001, 0.01) | 0.041 |
| ^*^Abbreviations: CI, confidence interval; FDR, false discovery rate. | | |  |
